# Salivary Total Antioxidant Capacity of Sportive Adolescents—The Effect of Antioxidant Vitamin Intake with Usual Diet and Physical Exercises

**DOI:** 10.3390/nu17223610

**Published:** 2025-11-19

**Authors:** Anna Gawron-Skarbek, Adam Marek Wróblewski, Jacek Chrzczanowicz, Dariusz Nowak, Tomasz Kostka

**Affiliations:** 1 Department of Geriatrics, Medical University of Lodz, 92-213 Lodz, Poland; tomasz.kostka@umed.lodz.pl; 2 Department of Medical Biochemistry, Medical University of Lodz, 92-215 Lodz, Poland; 3 Cardiac Rehabilitation Centre, Copernicus Memorial Hospital, 93-438 Lodz, Poland; jacekchrz@interia.pl; 4 Department of Clinical Physiology, Medical University of Lodz, 92-215 Lodz, Poland; dariusz.nowak@umed.lodz.pl

**Keywords:** total antioxidant capacity, saliva, DPPH, exercise, vitamin C, vitamin E, beta-carotene, young athletes

## Abstract

**Background:** The body requires effective antioxidant defense mechanisms to counter the effect of oxidative stress. The aim of the study was to evaluate the postprandial effect of antioxidative vitamin (C, E and β-carotene) consumption during breakfast and of aerobic exercise on salivary total antioxidant capacity (TAC). **Methods:** Fifty-one healthy male adolescents were examined (13–18 years; 15.4 ± 1.6). Dietary interviews including vitamin C, E, and β-carotene intake were performed twice, once on the examination day and again the day before. Salivary TAC was assessed using the DPPH method (2.2-diphenyl-1-picryl-hydrazyl) and expressed as % of free radical reduction. Saliva samples were assayed at three subsequent time-points: fasting (DPPH 1), after a meal—breakfast—(DPPH 2), and after aerobic exercise training (DPPH 3). **Results:** DPPH 2 was higher than DPPH 1 (16.8 ± 7.5 vs. 14.9 ± 7.2% of reduction; *p* = 0.03), and no differences were noted between DPPH 2 and DPPH 3 (16.8 ± 7.5 vs. 16.3 ± 6.5%; *p* > 0.05), nor between DPPH 1 and DPPH 3. Subjects with higher BMI demonstrated higher values of DPPH at all time-points of the study (*p* < 0.05). In turn, neither the DPPH values nor the changes in DPPH were related to weekly exercise-related energy expenditure (*p* > 0.05). No singular DPPH index was associated with the level of vitamin E or β-carotene intake with meals on the day before the study; however, DPPH 1 (rho = −0.38; *p* < 0.01) and DPPH 2 (rho = −0.45; *p* < 0.001) negatively correlated with vitamin C intake on the day before examination. **Conclusions:** In physically active adolescents, daily vitamin C consumption decreased salivary TAC, and the consumption of antioxidant nutrients/vitamins as part of a regular breakfast directly enhanced the antioxidant capacity of saliva; nevertheless, subsequent physical exercise had no detectable impact.

## 1. Introduction

Homeostasis between pro- and antioxidant compounds is maintained by the continual activity of a range of processes. The human body has a complex antioxidative defense system comprising three main lines of defense: free radical scavengers, antioxidant enzymes (e.g., glutathione peroxidase, superoxide dismutase and catalase), and the Fenton reaction system, which enables chelation of free metal ions. Free radical scavengers neutralize free radicals to become more stable radicals themselves. Moreover, vitamin C can regenerate oxidized antioxidant molecules [1].

The antioxidant defense system is affected by a number of factors, including physical effort [2], diet [3,4], smoking [5,6], health status/diseases [7,8], and applied pharmacotherapy. However, there is little concrete data regarding the influence of physical exercise on the antioxidant barrier and, as such, no formula aimed at improving antioxidant capacity currently exists [9,10,11,12]. Therefore, it is unclear whether enhancing antioxidant status would be more desirable for the body than maintaining the balance between oxidative stress and antioxidant potential.

Antioxidant capacity can be assessed by different methods, which achieve different results. Such methods include measuring the concentration or activity of singular antioxidants in body fluids (blood plasma/serum, saliva, urine, tears) or in cells/tissues, or by assessing Total Antioxidant Capacity/Status (TAC/TAS) [13,14,15]. Although TAC evaluation methods have certain limitations, e.g., sensitivity to only a defined group of antioxidants [16,17], this approach is widely used in experimental and clinical studies [15]. Recently, a promising new method for assessing TAC has been developed that could be useful in clinical studies. This method measures the total activity of circulating low-molecular-weight nonenzymatic antioxidants based on the ability of deproteinized body fluids (e.g., plasma, serum, and saliva) to decompose a 2.2-diphenyl-1-picryl-hydrazyl (DPPH) radical.

Human saliva is a mixture of gingival crevicular fluid (GCF), with a similar composition to plasma/serum, and it is the first line of defense against oxidative stress [14,18]. It includes a large number of organic and inorganic compounds which can be used as indicators of health status. As such, and due to its availability and noninvasive mode of collection, saliva could be used in diagnoses of pathological conditions/diseases in the oral cavity, as well as some systemic disorders [19]. For instance, salivary TAC is also known to be influenced by oral health status [20,21].

In the available literature there are no studies assessing simultaneously the impact of dietary antioxidants and exercise on salivary TAC in adolescents. Filling this gap of knowledge would enable better insight into planning these two basic non-pharmacologic behaviors in young people. Therefore, the aim of the study was to (1) evaluate the postprandial effect of consuming antioxidative vitamins (C, E, and β-carotene) as part of the typical breakfast, (2) measure the effect of aerobic exercise on salivary TAC, and (3) assess the relationship between the regular daily intake of antioxidative vitamins and the TAC of saliva in a group of healthy, young, physically active boys.

## 2. Materials and Methods

### 2.1. Subjects

The study included 83 healthy, physically active students in the Kazimierz Górski memorial sports school in Lodz (Poland). All were residents of the school dormitory, and were receiving their meals in the school canteen. The age range was 13–18 years, and the participants’ training experience ≥1 year; Physical Activity Level = 2.2 ÷ 2.4 [22]. Of these, 27 students were excluded from the study due to the following various reasons: absence from school, physical injury, lack of time in the second and/or in the third stage of the study, mild infection, or laboratory difficulties with saliva testing (i.e., presence of precipitates in the sample). Complete data was therefore collected from 56 adolescents. As only five female participants with complete data were enrolled, they were excluded from the study. Finally, 51 male students were included in the analysis (15.4 ± 1.6 years).

The whole study group was free from inflammation, chronic diseases (any medicaments were applied), and physical injury. The subjects were not overweight, obese (according to the BMI percentile chart for boys at age of 5 to 19 years), nor had an addiction history (tobacco, alcohol, and drugs). All students were provided with meals on a daily basis in the school canteen (institutional food). None used any special diet. The study protocol was approved by the local ethics committee (RNN/15/15/KE/L) and informed consent was obtained from parents or legal guardians.

### 2.2. Protocol and Measures

The examinations took place in the school. The laboratory measurements were performed in the Department of Clinical Physiology, Medical University of Lodz, and the dietary intake was analyzed in the Department of Hygiene and Health Promotion, Medical University of Lodz. The students were asked to report to the school hall, after overnight fasting, between 7:00 and 7:45 a.m. They were also asked not to brush their teeth before sample collection, just to slightly gargle the oral cavity with water (time window between tooth cleaning and saliva collection was ±10 h).

An unstimulated saliva sample was given by each student and reading pH 1 was assayed immediately. Following this, the subjects were interviewed for demographic and behavioral features related to dietary habits, physical activity, oral hygiene, smoking, and drug use; the data was collected using a questionnaire designed for the study based on WHO CINDI recommendations [23]. Anthropometric and blood pressure measurements were performed, and a 24 h dietary recall from the day before an examination was obtained from each individual.

Next, the subjects consumed their breakfast at the school canteen. A second saliva sample was then collected within an hour, before their usual exercise training, and reading pH 2 was tested. Then the dietary intake from breakfast was recorded and the students went to their training. Each of the respondents, both the day before and on the day of the study, could eat what the kitchen offered in any amount according to their usual preferences.

After aerobic exercise (physical activity intervention) the third saliva sample was collected and pH 3 was recorded. A single training unit lasted 1.5 h and consisted of three parts: a warm-up (±20 min, including jogging, jumping, stretching, and activities reflecting the type of movements/actions during the sport events), a main training session with discipline-specific exercises (±60 min), and a cool down (±15 min, including stretching of muscle groups loaded during a training session). Exercise training was the same for all the participants.

#### 2.2.1. Anthropometric and Blood Pressure Data

Height and weight were measured and Body Mass Index (BMI; kg∙m^−2^) was calculated [24]. Waist and hip circumference measurements were taken, and Waist-to-Hip Ratio (WHR) was computed as an index of visceral obesity. An electronic manometer with oscillometric technique (UA-767PC) was used to measure systolic (SBP) and diastolic blood pressure (DBP), and heart rate (HR) at rest [25].

#### 2.2.2. Energy Expenditure

The exercise-related energy expenditure (kcal⋅week^−1^) was calculated based on the time (hours and minutes) scheduled for recreational/sports physical activities. The calculation was performed according to Fox et al. [26].

#### 2.2.3. Nutritional Assessment

Dietary recall was supported using an album of photographs of food products and dishes from the National Food and Nutrition Institute (NFNI) in Warsaw [27]. The Diet 5.0 software (license No: 52/PD/2013), also designed by the NFNI, was used to assess nutrient, vitamin, mineral, and energy intake in the daily food rations (DFR) [22,28].

#### 2.2.4. Salivary TAC and pH

Before the unstimulated saliva samples (±5 mL) were secured [29], pH assays were performed with test paper (Whatman, Maidstone, UK). In the laboratory, the samples were centrifuged to separate all debris (10,000 rpm, 10 min, 4 °C). The supernatant was stored at −80 °C until the experiment, but not longer than 30 days.

Salivary TAC was assessed using the spectrophotometric (Ultrospec III with Spectro-Kinetics software—LKB Biochrom Pharmacia, Cambridge, UK) DPPH test, as described previously [15,30,31]. The analysis comprised samples of saliva collected at three subsequent time-points: fasting (DPPH 1), after a meal—usually breakfast—(DPPH 2), and after aerobic exercise intervention (DPPH 3). For singular DPPH determination, 25 μL of deproteinized saliva was added to 975 μL of DPPH reagent mixture. To enhance the data reliability, all individual results were calculated as a mean, from three separate experiments (expressed as % of DPPH reduction). The measurement procedures were performed by a qualified laboratory technician who was blinded to the participant qualification criteria and the time-point of the analyzed samples.

### 2.3. Statistical Analysis

Data were verified for the normality of distribution using the Shapiro–Wilk test, and homogeneity of variance using Levene’s test. Variables that did not meet the assumption of normality were analyzed with non-parametric statistical tests. DPPH and pH values between the three time-points were compared using the T-test for dependent samples and the Wilcoxon matched-pairs test. The results for continuous variables were presented as mean ± SD and median (Q1–Q3). The Spearman rank correlation with 95% confidence intervals (CI) was used to determine the relationships between the selected numerical variables. Effect sizes based on Cohen’s d were calculated. An effect size of 0.2 to <0.5 and ≥0.5 to <0.8 has been suggested to represent a small and medium effect, respectively, while an effect size ≥ 0.8 represents a large effect. The level of statistical significance was set at *p* < 0.05. Statistica version 13 was used (StatSoft Polska Sp. z o. o., Kraków, Poland).

## 3. Results

### 3.1. General Characteristics

Detailed demographic, anthropometric resting blood pressure and energy expenditure data are presented in Table 1. The entire study group was within the normal BMI range and did not have high blood pressure.

### 3.2. Nutrient Intake

Table 2 presents data regarding energy and the consumption of various DFR nutrients with meals on the day before the study and with the breakfast on the day of the study; Table 3 presents the intake of the antioxidant vitamins C, E, and β-carotene.

### 3.3. Analyses of DPPH Changes and Selected Correlates

**Figure 1:**
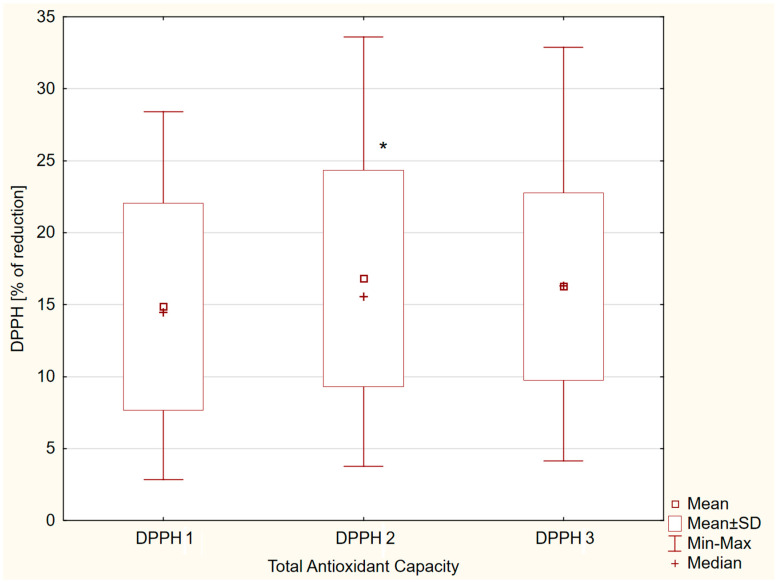
Salivary DPPH values: fasting (DPPH 1), after dietary intake (DPPH 2), and after aerobic exercise intervention (DPPH 3) in group of male adolescents. *—*p* < 0.05.

A significant difference was observed between DPPH 1 and DPPH 2, i.e., the DPPH values increased after eating a meal (potential antioxidant effect of vitamin C, E, and β-carotene intake): 14.9 ± 7.2%; 95% CI = 12.8–16.9% vs. 16.8 ± 7.5%; 95% CI = 14.7–18.9%; *p* = 0.03. However, the level of DPPH was not affected by exercise (DPPH 1 vs. DPPH 3: 14.9 ± 7.2%; 95% CI = 12.8–16.9% vs. 16.3 ± 6.5%; 95% CI = 14.4–18.1; *p* > 0.05) (Figure 1).

**Figure 2:**
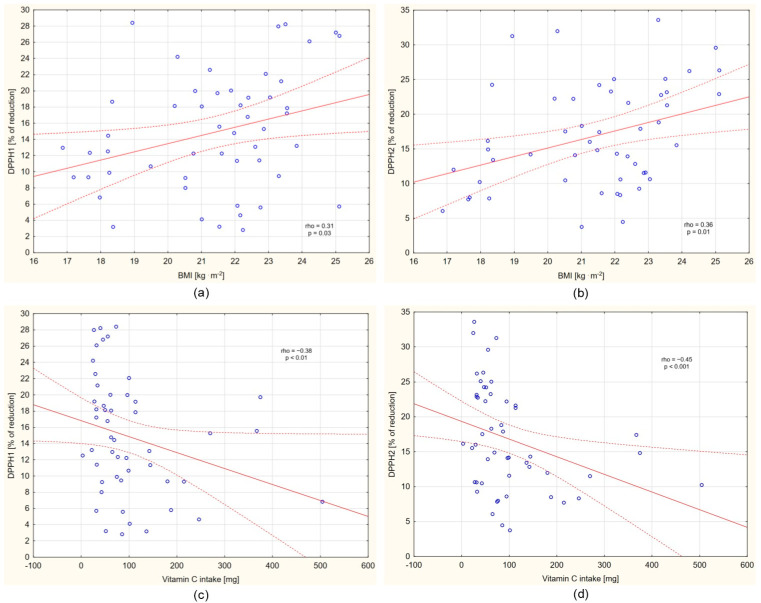
Correlations between DPPH and BMI (**a**,**b**), and between DPPH and vitamin C intake on day before the examination (**c**,**d**).

Higher DPPH 1 (rho = 0.31; 95% CI = 0.03–0.54; *p* = 0.03), DPPH 2 (rho = 0.36; 95% CI = 0.09–0.59; *p* = 0.01), and DPPH 3 (rho = 0.31; 95% CI = 0.03–0.55; *p* = 0.03) were noted in subjects with higher BMI values (Figure 2a,b; small effect size). In turn, neither the DPPH values nor the changes in DPPH (neither ΔDPPH 3–DPPH 1, nor ΔDPPH 3–DPPH 2) were associated with exercise-related energy expenditure (*p* > 0.05).

No singular DPPH index was associated with the level of vitamin E or β-carotene intake with meals on the day before the study; however, DPPH 1 (rho = −0.38; 95% CI = −0.60–−0.11; *p* < 0.01) and DPPH 2 (rho = −0.45; 95% CI = −0.65–−0.19; *p* < 0.001) negatively correlated with vitamin C intake (Figure 2c,d; small effect size). Individuals with higher vitamin C intake demonstrated lower fasting and postprandial salivary TAC. Vitamin C consumed with meals the day prior to the study had no effect on DPPH 3.

Meanwhile, the level of vitamins C, E, and β-carotene consumed with breakfast on the day of the examination was not related to either DPPH 2 or DPPH 3, nor to a change in DPPH (ΔDPPH 2–DPPH 1).

## 4. Discussion

Few reports have examined the antioxidant capacity of saliva in adolescents, especially interventional studies [32]. As such, the present study is one of the few to use saliva to determine the effect of physical activity and the amount of antioxidant vitamins consumed with a meal (not as supplements) on TAC (DPPH) levels. It was observed that salivary TAC of the studied active male adolescents significantly increased after breakfast, which could be related to the consumption of antioxidant vitamins contained in a meal. We may suppose that the observed postprandial increase in TAC could be related also to non-vitamin components of meals—to the polyphenols contained, for example, in tea, fruit, and vegetables—or perhaps also to the sources of fructose (fruit), or purine rings (meat), as substrates for future uric acid synthesis. In contrast, the aerobic exercise intervention had no influence on salivary TAC, as indicated by DPPH testing, in a relatively short period after the intervention.

These results are partially consistent with those of a previous study in a group of older adults, which showed that, unlike supplementation, higher habitual dietary intake of vitamin C corresponds to lower salivary TAC when measured via FRAS (Ferric Reducing Ability of Saliva), but neither vitamin C, E, nor β-carotene affected salivary TAC when assessed via DPPH [33]. A recent systematic review suggested that increased dietary antioxidant intake is more closely linked to enhanced salivary TAC in different age groups [34,35]. Kamodyová et al. report that a high single intake of a 250 mg dose of vitamin C augmented salivary TAC and ferric reducing antioxidant power (FRAP) in healthy subjects (*n* = 19) [36]. Although the intake of vitamins C, E, and β-carotene with meals was not as high in the current study, the effect on salivary TAC was still present.

The studied adolescents did not consume a vitamin-rich diet, but it seems that this was enough to improve the antioxidant capacity of saliva. It should be noticed that some data variability, as the intake of vitamin C, vitamin E, and β-carotene had a high variability among participants, could be related to the differentiated dietary preferences of youth—some of them did not like vegetables or fruits, and others quite the opposite. Interestingly, our findings indicate negative correlations between the level of vitamin C intake from a 24 h interview and either DPPH 1 or DPPH 2 in the study group. Adolescents whose daily vitamin C intake was higher the day before the study showed lower fasting and postprandial antioxidant capacity of saliva on the next day. However, this relationship was no longer identified after exercise. This could mean that although the postprandial antioxidant effect in saliva occurs, it may be short-lived, and that after a longer break from the meal, the antioxidant effect in saliva is counterbalanced by habitual vitamin C intake. It may be the case that this is intended to increase the level of antioxidant defense in the oral cavity in saliva. Several existing studies indicate pro-oxidative properties of vitamin C at low concentrations (“second face” of vitamin C), especially in the presence of interlocutory metal ions (Fe and Cu), which may putatively attenuate the antioxidant defense system [37,38,39,40]. Interestingly, the bioavailability of vitamin C/ascorbic acid is dose-dependent, i.e., higher absorption is observed at lower doses, and it is actively transported through cell membranes.

The amount of serum vitamin C can be modified in relation to dietary supply by active vitamin C transporters (SVCT1 and SVCT2) [41]. Hence, the vitamin C level in saliva could be influenced by its absorption with previous meals, thus determining the level of salivary TAC.

It seems that salivary TAC may be vulnerable to antioxidant intake in a time-dependent and antioxidant-specific manner. Recent (breakfast) intake may increase TAC while more remote (the day before) intake may be related to lower TAC, but only in relation to vitamin C intake and not to vitamin E or β-carotene intake. However, considering the contradictory results of previous research, which suggests an indirect relationship between antioxidant vitamin intake and TAC, and the possible contribution of other factors, further analyses are required to better understand the nature of the free radical scavenging mechanisms used in the body.

On the other hand, the meta-analysis of 22 studies indicated an increase in salivary TAC despite the oxidative stress linked to dental caries, implying the existence of a compensatory mechanism activated when necessary [20]. Another meta-analysis showed attenuated TAC in plasma, serum, and GCF in periodontitis; however, salivary TAC was unchanged [21]. Thus, further research should focus on a comprehensive assessment of the body’s oxidative status (e.g., plasma, GCF, and cellular oxidative status of adjacent tissues) to identify the potential antioxidative reservoir utilized to counterbalance salivary oxidative stress on demand.

The following certain factors may have a significant influence on the effect of physical exercise on saliva TAC level: the time of sample measurement (in our case, immediately after the end of training), the type of exercises (aerobic or anaerobic), and its duration and intensity. In the present study, no significant increase in salivary TAC was observed after aerobic exercise, which may indicate that the physically active adolescent boys have more advanced adaptive capacities for physical effort than typical adolescents, at least in saliva. This higher level of exercise tolerance may have resulted from their everyday sporting school activities; however, exercise-related energy expenditure was not found to influence TAC/DPPH index in saliva.

Grzesiak-Gasek et al. report an increase in salivary TAC in thirty young (13–16 years) swimmers after swimming training, which they attribute to an adaptive mechanism or a defensive reaction to increased oxidative stress induced by physical exercise. After resting, salivary TAC level decreased but remained higher than at rest [42]. Also, González et al. [2] indicate increased TAC in healthy subjects immediately after aerobic exercise (after 10,000 m race) and a decrease in the salivary marker of oxidative stress (lipid hydroperoxide).

In contrast to our present research, Mahdivand et al. report a decrease in TAC (assessed by FRAP) both immediately after and 24 h after a single session of combined strength and endurance training in a group of 20 male athletes [43]. In turn, Deminice et al. [44] note that salivary uric acid, a main endogenous antioxidant, increased post-acute resistance training and that salivary uric acid correlated with blood serum acid. Further studies are needed to assess TAC simultaneously in saliva and other fluids, such as blood plasma, to confirm the presence of systemic antioxidative mechanism homeostasis among well-trained adolescents.

However, due to the small number of similar studies, it is difficult to relate our present results to the wider literature. Nevertheless, Pani et al. [45] reported a significantly lower reduction in salivary TAC in dental students engaging in high levels of exercise compared to their counterparts from a low-exercise group, during examination week. This supports the theory that more physically active people have better adaptation mechanisms. They conclude that regular exercises may protect against the oxidative stress associated with academic stress. Additionally, Munther et al. also confirm that total salivary antioxidant activity was higher in men who exercised compared to those who did not, regardless of smoking [46].

In contrast, taekwondo athletes were found to have a markedly suppressed salivary antioxidant capacity immediately after a training session, though it quickly returned to pre-exercise values [10], regardless of whether green tea or water was consumed by subjects. Similarly, exhaustive aerobic exercise was found to induce oxidative stress in the saliva of young girls, which affected TAC levels (DPPH) [47]. Furthermore, Babaei et al. observed that acute physical exercise contributed to a decrease in salivary vitamin C concentration in sedentary men, which may have impacted antioxidant capacity [48]. These examples illustrate the complex relationship between physical exercise and salivary TAC according to type of physical activity (aerobic/anaerobic), its intensity, or one’s level of proficiency in sport. A systematic review, by Alves et al., of the effects of physical exercise and its accompanying changes in salivary oxidative stress/antioxidant capacities also concluded that the wide heterogeneity of study methodology leads to divergent data [49].

An interesting additional observation was the identification of positive correlations between DPPH 1, DPPH 2, and DPPH 3, and BMI. At each salivary TAC measurement time-point, a higher antioxidant capacity of saliva indicated a better, but not impaired, nutritional status, as assessed by BMI. Unfortunately, few papers describe the relationship between salivary TAC and nutritional status in young subjects with normal weight (no overweight/obesity); however, a study of salivary TAC in a group of thirty-year-old participants by Safabakhsh et al. did not identify significant differences between the obese and normal-weighted individuals [50]. On the other hand, Gunjalli et al. reported higher salivary TAC in overweight and obese 6- to 12-year-old children in India compared to their slim counterparts. The authors note that these children belong to a high socioeconomic class and attribute the outcome to a diet rich in phytonutrients and antioxidants [51]. Also, Zalewska et al. report that overweight and obese adolescents (aged 11–18) demonstrate stronger antioxidant barrier in saliva (higher TAC) and higher concentrations of individual antioxidants, such as uric acid or enzymes, superoxide dismutase, catalase, and peroxidase, compared to controls [52]. Based on these findings and our present results, it can be assumed that a poorer nutritional status, but not being underweight, may translate into the weaker antioxidant capabilities of saliva; however, this hypothesis requires additional research, ideally in groups without any accompanying health problems.

The study does have some limitations. For example, it would have benefited from a larger sample size. In addition, regarding recruitment, the subjects were included based on the typical time period that boys enter puberty in Poland; this is currently believed to be around 13–14 years of age. However, no assessment of the stage of the development of each participant was made, which could influence the level of the antioxidant adaptive mechanisms of saliva. The puberty period may affect the antioxidative activity of saliva [8] but, for example, in the study by Esenlik et al. [53], on patients with fixed orthodontic appliances, no difference was found regarding the antioxidant status of saliva between the pubertal and postpubertal groups. Future studies should assess the potential impact of the stage of puberty on salivary antioxidant status. Due to the small number of girls who agreed to participate in the project (i.e., five participants), the study included only male individuals, which weakens the possibility of extrapolating the results to the entire population. Further research should be aimed at female athletes and adolescents in other sports disciplines to permit further generalization. Furthermore, the DPPH method itself is limited in that it omits protein antioxidants (enzymes, albumins, and glutathione) which might also affect the final results; in addition, it cannot be excluded that the DPPH effect was local rather than systemic, and greater changes in saliva could occur later in the oral cavity rather than in the circulation (in blood plasma).

Nevertheless, the study also has a number of strengths. The subject selection allowed the formation of a homogeneous group consisting of healthy, sportive young male adolescents with similar anthropometric and dietary characteristics: all subjects were engaged in a similar lifestyle and ate meals served by the same canteen. In addition, the group was not subject to possible confounding factors, i.e., no participants smoked, or were overweight or obese. Our results also provide an opportunity to identify some nutritional shortcomings in the diet of young athletes fed by the mass catering system. Still, further studies are needed to confirm these findings and to shed more light on the habitual antioxidant vitamin intake- and physical exercise-related modulation of salivary TAC.

## 5. Conclusions

In conclusion, in physically active adolescents, daily vitamin C consumption decreased salivary TAC, and the consumption of antioxidant nutrients/vitamins as part of a regular breakfast directly enhanced the antioxidant capacity of saliva. In addition, subsequent physical exercise had no detectable impact; however, the potential systemic impact of physical training cannot be excluded. Despite limited evidence, salivary TAC seems to be a promising index for assessing biochemical changes in the body caused by behavior-dependent factors.

## Figures and Tables

**Table 1:** Baseline characteristics of the study group.

Parameter	Mean ± SD Median (Q1–Q3)
Age [years]	15.4 ± 1.6 16.0 (14.0–17.0)
Height [m]	1.76 ± 0.1 1.76 (1.69–1.82)
Body Mass [kg]	66.8 ± 12.4 68.0 (60.0–76.0)
Body Mass Index [kg∙m^−2^]	21.4 ± 2.2 21.9 (20.2–22.9)
Waist Circumference [cm]	72.2 ± 5.6 72.0 (70.0–75.0)
Hip Circumference [cm]	89.5 ± 8.2 90.0 (82.0–95.0)
Waist-to-Hip Ratio	0.81 ± 0.05 0.81 (0.78–0.83)
Systolic Blood Pressure [mmHg]	124.6 ± 12.8 124.0 (116.0–131.0)
Diastolic Blood Pressure [mmHg]	68.6 ± 9.8 68.0 (62.0–75.0)
Heart Rate [beats per minute]	68.6 ± 9.8 68.0 (62.0–75.0)
Exercise-related Energy Expenditure [kcal∙week^−1^]	7000 ± 1804 6300 (6000–8400)

**Table 2:** Dietary characteristics of the study group based on a 24 h dietary recall and from the breakfast on a study day.

Parameter	24 h Mean ± SD Median (Q1–Q3)	Breakfast Mean ± SD Median (Q1–Q3)
Total energy [kcal∙d^−1^]	3008 ± 880 3106 (2317–3434)	756 ± 588 610 (554–720)
Proteins [g]	104.4 ± 32.0 103.5 (86.1–124.6)	22.1 ± 9.8 20.6 (16.4–25.6)
Animal proteins [g]	61.4 ± 25.4 60.6 (44.4–78.1)	11.2 ± 7.8 10.6 (6.2–13.6)
Plant proteins [g]	42.6 ± 11.1 42.5 (35.8–48.4)	11.0 ± 3.7 10.2 (9.7–12.4)
Carbohydrates [g]	453.9 ± 136.0 456.5 (380.7–522.2)	92.2 ± 31.9 78.8 (74.4–114.6)
Absorbable carbohydrates [g]	429.6 ± 131.9 437.8 (360.6–495.0)	88.6 ± 31.1 73.4 (71.1–110.3)
Sucrose [g]	111.3 ± 62.9 105.8 (79.8–132.9)	11.0 ± 7.9 7.9 (6.4–13.5)
Fiber [g]	24.4 ± 7.9 23.5 (18.4–29.5)	3.7 ± 2.2 2.7 (2.2–4.3)
Fats [g]	95.0 ± 39.9 96.1 (67.3–115.8)	34.8 ± 62.4 22.3 (18.2–27.6)
Saturated fatty acids [g]	40.7 ± 17.4 40.6 (27.8–50.7)	19.4 ± 39.4 12.3 (8.9–16.4)
Monounsaturated fatty acids [g]	36.6 ± 17.9 34.0 (24.1–45.2)	10.7 ± 17.8 7.5 (5.3–8.4)
Polyunsaturated fatty acids [g]	11.1 ± 4.9 10.8 (7.2–12.8)	2.6 ± 3.7 1.8 (1.6–2.2)
Cholesterol [mg]	322.8 ± 137.7 298.1 (241.3–362.3)	102.9 ± 185.2 68.6 (44.2–88.8)

**Table 3:** Vitamin C, vitamin E, and β-carotene intake with daily food rations on the day before the study, based on a 24 h dietary recall, and with breakfast on the day of the study, in a group of adolescents.

Parameter	24 h Mean ± SD Median (Q1–Q3)	Breakfast Mean ± SD Median (Q1–Q3)
Vitamin C [mg]	100.5 ± 99.2 69.0 (39.8–113.4)	6.3 ± 7.6 2.5 (0.0–10.2)
Vitamin E [mg]	8.8 ± 4.2 8.0 (6.2–10.3)	2.1 ± 3.7 1.3 (1.1–1.7)
β-carotene [µg]	4757 ± 5968 3355 (1283–5658)	192 ± 298 111 (76–192)

## Data Availability

The data presented in this study are available on request from the corresponding authors. The data are not publicly available to preserve privacy of minor participants.
